# Pine Defensive Monoterpene α-Pinene Influences the Feeding Behavior of *Dendroctonus valens* and Its Gut Bacterial Community Structure

**DOI:** 10.3390/ijms17111734

**Published:** 2016-11-01

**Authors:** Letian Xu, Zhanghong Shi, Bo Wang, Min Lu, Jianghua Sun

**Affiliations:** 1 State Key Laboratory of Integrated Management of Pest Insects and Rodents, Institute of Zoology, Chinese Academy of Sciences, No. 1 Beichen West Road, Chaoyang District, Beijing 100101, China; letian0926@163.com (L.X.); sunjh@ioz.ac.cn (J.S.); 2 TEDA Institute of Biological Sciences and Biotechnology, Nankai University, TEDA, Tianjin 300457, China; 3 Key Laboratory of Molecular Microbiology and Technology, Ministry of Education, Tianjin 300071, China; 4 Fujian Provincial Key Laboratory of Insect Ecology, College of Plant Protection, Fujian Agriculture and Forestry University, Fuzhou 350002, China; shizh@fafu.edu.cn; 5 Key Laboratory of Tropical Forest Ecology, Xishuangbanna Tropical Botanical Garden, Chinese Academy of Sciences, Menglun 666303, China; vincentwb@126.com

**Keywords:** *Dendroctonus valens*, α-pinene, gut microbiota

## Abstract

The exposure to plant defense chemicals has negative effects on insect feeding activity and modifies insect gut microbial community composition. *Dendroctonus valens* is a very destructive forest pest in China, and harbors a large diversity and abundance of gut microorganisms. Host pine defensive chemicals can protect the pines from attack by the holobiont. In this study, boring length of *D. valens* feeding on 0 mg/g α-pinene and 9 mg/g α-pinene concentration in phloem media for 6 and 48 h were recorded, and their gut bacterial communities were analyzed in parallel. Nine milligram per gram α-pinene concentration significantly inhibited boring length of *D. valens* and altered its gut microbial community structure after 6 h. The inhibition of boring length from 9 mg/g α-pinene in diets ceased after 48 h. No significant differences of the bacterial communities were observed between the beetles in 0 and 9 mg/g α-pinene concentration in phloem media after 48 h. Our results showed that the inhibition of the feeding behavior of *D. valens* and the disturbance to its gut bacterial communities in 9 mg/g α-pinene concentration in phloem media after 6 h were eliminated after 48 h. The resilience of gut bacterial community of *D. valens* may help the beetle catabolize pine defense chemical.

## 1. Introduction

Gut microbiota are ubiquitous for insect herbivores and are often critical to insect biological performance and ecological functioning, such as augmentation of nutritional availability [1,2,3], protection of insects from pathogens and parasites [4,5], facilitation of pheromone production [6,7], and detoxification of harmful toxins produced by the host plant defense system [8,9]. The gut microbiome has also been recognized as a major force in shaping insect-plant interactions [10,11,12]. In addition to degrading plant compounds that would supplement missing nutrients [13], these microbial symbionts are capable of tolerating and detoxifying defensive chemicals of host plants [9,14,15]. On the contrary, the defensive chemicals of host plants along with decreased nutrition adversely affect insects’ growth and development [16], which can influence herbivore’s gut microbial community compositions when herbivores feed on plant tissues [17,18,19].

Bark beetles (Curculionidae: Scolytinae) are known as the most damaging pests of coniferous forests worldwide, which has caused extensive conifer tree mortality and economic damage [20,21,22,23]. Conifers are able to defend themselves against attacks with various resistance mechanisms [24], e.g., elevated levels of induced defensive chemicals, including monoterpenes, which pose significant barriers to the beetles’ utilization of plant substrates [25,26,27,28]. In addition, the defensive chemicals influence bark beetles’ associated microorganisms including growth of vectored fungi [29,30], and survival of their associated bacteria [9,31]. Some of these microbes can assist bark beetle-microorganisms holobiont to degrade or metabolize the toxic chemicals of host plants [15,32], while others help to facilitate the beetles’ pheromone production ability [7], and thus have vital ecological functions for the adaptation and reproduction of beetles. Furthermore, at the community level, a metagenome-wide study of associated microbiota of *Dendroctonus ponderosae* showed that many genes belonging to several genera are involved in defensive terpene degradation [33].

The red turpentine beetle (RTB), *Dendroctonus valens* LeConte (Scolytinae), is a very destructive pest in China, and was introduced to China in the early 1980s from North America and has caused mortality of millions of healthy pine trees [23,34]. The diversity of the gut bacteria has been well investigated using culture dependent method, and some of the gut bacteria have been proven to simultaneously help produce the pheromone verbenone of *D. valens* and degrade 20%–50% host defensive chemical α-pinene [7,9]. Meanwhile, the tolerance of three *D. valens* gut bacteria to different concentrations of α-pinene are species-dependent [9]. Although we know the toxic activities of α-pinene to both *D. valens* and its gut bacteria [9,35,36], the influence of α-pinene on the feeding behavior of *D. valens* and its gut bacterial community structure in vivo is little known. The goal of this study was to study how pine defensive chemical α-pinene influences feeding behavior of *D. valens*, and to further test whether the chemical influences its gut bacterial community structure.

## 2. Results

### 2.1. The Influence of α-Pinene on the Boring Length of Dendroctonus valens

**Figure 1:**
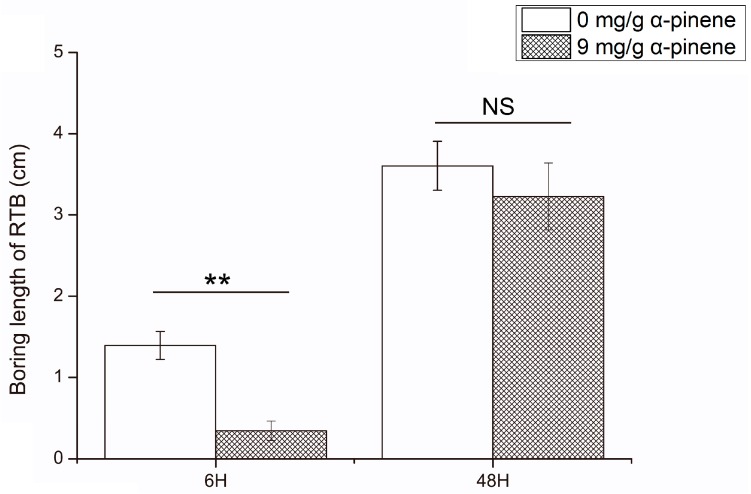
The boring length (Mean ± SEM) of *Dendroctonus valens* feeding in 0 and 9 mg/g α-pinene phloem media after 6 h and 48 h. The data were analyzed using independent *t*-test or Mann–Whitney *U*-test depending on the results of the test of normality and homogeneity of variance. Asterisks indicate a statistically significant difference (** *p* < 0.01); NS, not significant; RTB, red turpentine beetle.

The boring length of *D. valens* in 9 mg/g α-pinene 6 h feeding group was significantly less than those in 0 mg/g α-pinene 6 h feeding group (Mann–Whitney *U*-test, *p* < 0.05, Figure 1). There were no significant differences of boring length of *D. valens* between 0 mg/g α-pinene 48 h feeding group and 9 mg/g α-pinene 48 h feeding group.

### 2.2. Alpha Diversity

**Figure 2:**
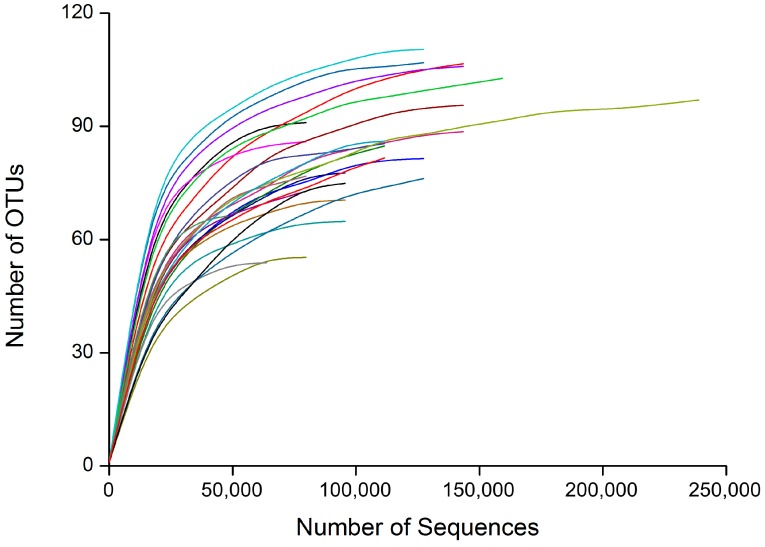
Rarefaction curves of the 20 samples (different color lines) based on MiSeq sequencing of bacterial communities. OTUs, Operational Taxonomic Units.

In the 25 representative gut samples of *D. valens* from four different treatments, we obtained a total of 2,614,017 sequences (99.8% of the total trimmed 2,618,895) and grouped into 2722 Operational Taxonomic Units (OTUs) at 97% similarity cut-off level. Rarefaction analysis showed that the OTUs of 25 samples gradually increased and then reached stable values with the increase of the number of measured sequences, which indicate that most bacterial sequences obtained by the MiSeq sequencing system reflected the abundance and diversity of the microbiota (Figure 2). Alpha diversity was estimated by five indices including number of OTUs, ACE, Chao1, Shannon, and Simpson. There were no significant differences among four groups in three diversity indices (Number of OTUs, Chao1 index, ACE index) (Table 1). Shannon diversity index of the samples in 9 mg/g α-pinene 48 h feeding group was significantly lower than the index of other three feeding groups (Table 1; *F*_3,21_ = 4.31, *p* < 0.05). Simpson diversity index of the samples in 9 mg/g α-pinene 6 h feeding group was significantly higher than the index in 0 mg/g α-pinene 6 h feeding group (Table 1; *F*_3,21_ = 16.07, *p* < 0.05).

### 2.3. Principal Component Analysis of the Gut Microbiota of D. valens

**Figure 3:**
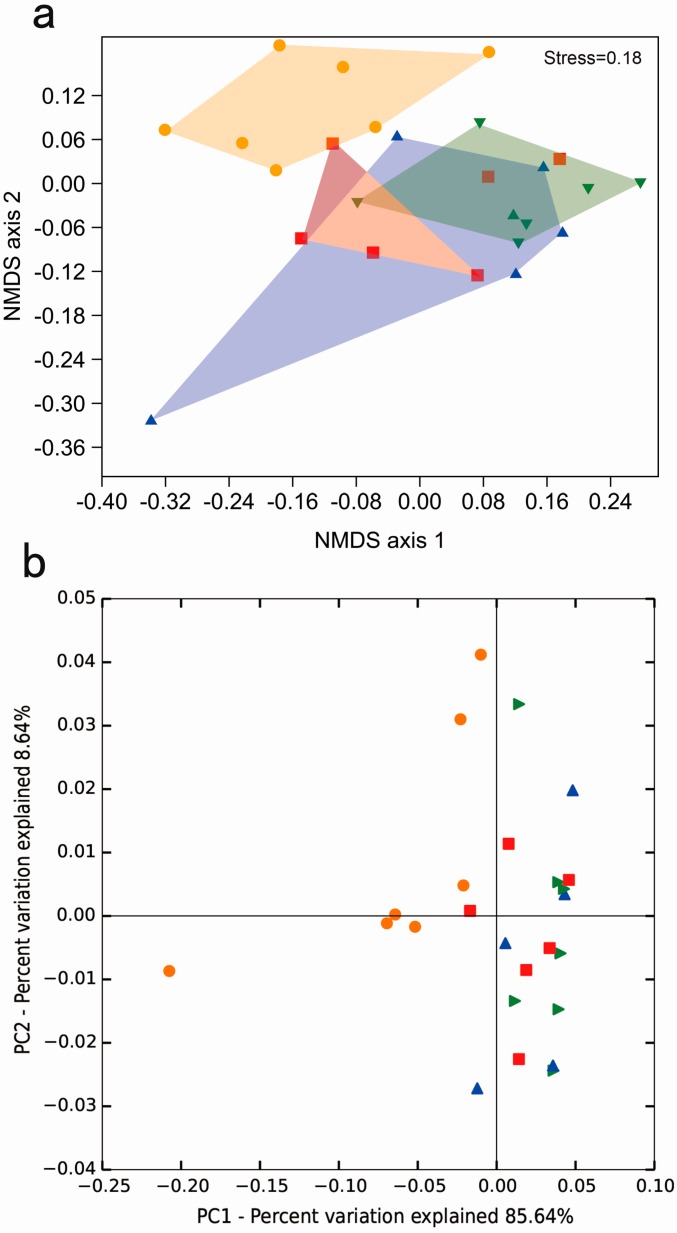
Principal component analysis of all bacterial communities. (**a**) Non-metric multidimensional scaling (NMDS) diagrams of 25 samples, based on Jaccard distance matrix for bacterial communities that consisted of OTUs (97% similarity level). Bacterial communities of samples in 9 mg/g α-pinene 6 h feeding group were significantly separated from the other three feeding groups (ANOSIM, *p* < 0.01); (**b**) Principal coordinate analysis (PCoA) plots based on the weighted UniFrac metric for bacterial communities. Permutational multivariate analysis of variance indicated the bacterial community of samples in 9 mg/g α-pinene 6 h feeding group was significantly different with the other three feeding groups (PERMAOVA, *p* = 0.001). The red square represents samples in 0 mg/g α-pinene 6 h feeding group, orange circle represents samples in 9 mg/g α-pinene 6 h feeding group, blue triangle represents samples in 0 mg/g α-pinene 48 h feeding group, and green triangle represents samples in 9 mg/g α-pinene 48 h feeding group.

Based on the detected OTUs across the 25 samples, an non-metric multidimensional scaling (NMDS) ordination analysis result showed that *D. valens* gut bacterial communities in 9 mg/g α-pinene 6 h feeding group clustered independently and distinctly from other three feeding groups (0 mg/g α-pinene 6 h, 0 mg/g α-pinene 48 h, and 9 mg/g α-pinene 48 h), which were similar to each other, and the result was confirmed in the NMDS diagram using the Jaccard similarity metric (Figure 3a; analysis of similarities (ANOSIM), *p* < 0.001). Community compositions of gut samples in 9 mg/g α-pinene 6 h feeding group were significantly different from those gut samples in 0 mg/g α-pinene 6 h, 0 mg/g α-pinene 48 h, and 9 mg/g α-pinene 48 h feeding groups (Table S1, ANOSIM, *p* < 0.05), and no statistical difference exist in bacterial community composition of *D. valens* gut samples among 0 mg/g α-pinene 6 h, 0 mg/g α-pinene 48 h, and 9 mg/g α-pinene 48 h feeding groups (Table S1). The phylogeny-based weighted UniFrac principal coordinate analyses considering relative abundances of OTUs showed similar results, which was further corroborated by a dissimilarity test PERMANOVA (Figure 3b; PERMANOVA, *p* < 0.05).

### 2.4. The Analysis of Community Composition at Genus Levels

**Figure 4:**
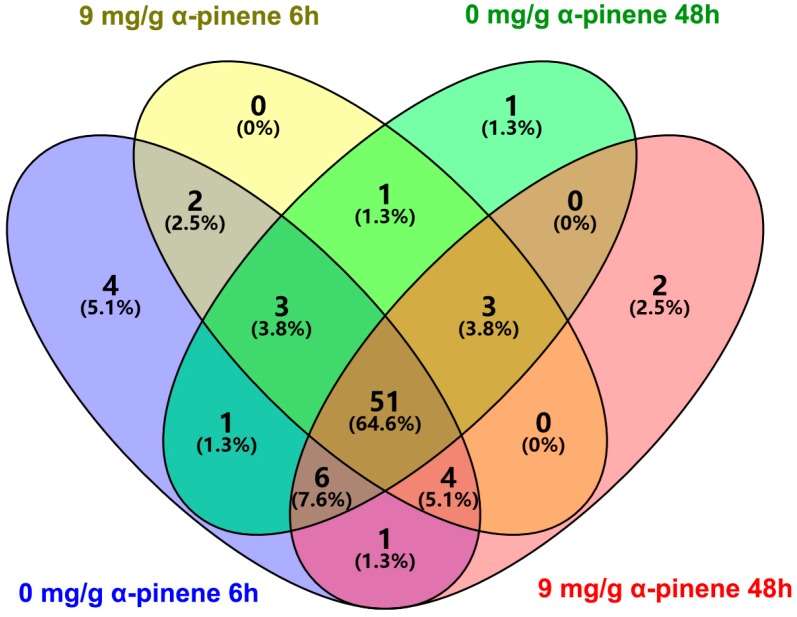
Venn diagram (at distance 0.03) showing the shared and unique genera between 0 mg/g α-pinene 6 h, 9 mg/g α-pinene 6 h, 0 mg/g α-pinene 48 h, and 9 mg/g α-pinene 48 h feeding groups.

The sequences could be assigned to 79 genera and their abundance are shown in Table S2, and a total of 72 genera was shared by four different groups (Figure 4), which account for 98.66%–99.87% of the total sequences in the respective samples (Table S2). The genera with an abundance of at least 0.1% of the total number of reads in at least one sample are presented in Table 2. The gut samples in 9 mg/g α-pinene 6 h feeding group has a significantly higher proportion of *Erwinia* spp. and significantly lower proportion of *Sphingomonas* spp. than samples in 0 mg/g α-pinene 6 h, 0 mg/g α-pinene 6 h, and 9 mg/g α-pinene 48 h feeding groups (Table 2; *Erwinia*, One-way ANOVA, *F*_3,21_ = 9.02, *p* < 0.05; *Sphingomonas*, One-way ANOVA, *F*_3,21_ = 8.89, *p* < 0.05). The proportion of genera *Burkholderia* in gut samples of 9 mg/g α-pinene 6 h feeding group was significantly lower than those in gut samples of 9 mg/g α-pinene 48 h feeding group (Table 2; One-way ANOVA, *F*_3,21_ = 3.54, *p* < 0.05).

## 3. Discussion

The toxic activities and the influence of host defensive chemicals on bark beetles’ associated microbes at species level have already been investigated [9,31,37]. Host defensive chemical α-pinene has been shown to attract bark beetles [28,38], and is toxic to beetles at high concentration [25,28,36]. Some studies showed that α-pinene could be converted to verbenol which is a precursor chemical of *D. valens* verbenone pheromone [7,39]. The current study evaluated the influence of chemical defensive chemical α-pinene on the gut microbiota of Chinese *D. valens* in vivo using high throughput pyrosequencing approach, and results showed that the concentration of host defensive monoterpene α-pinene in diets altered the gut bacterial community of *D. valens* (Figure 3). Specifically, the gut bacterial communities of *D. valens* in the 9 mg/g α-pinene 6 h feeding group were significantly different in the beetles feeding on 0 mg/g α-pinene phloem media after 6 h (Figure 3). This suggests that high concentrations of α-pinene are capable of altering the community structure of *D. valens* gut microbiota within a short time period (6 h). Similar results have shown that plant defensive chemicals influence herbivore insect gut microbiota in other systems, e.g., aspen defense chemicals were reported to influence the midgut bacterial community composition of *Lymantria dispar* L. [19,40], and the gut microbial community structures of *Neotoma bryanti* and *Neotoma lepida* were altered by plant secondary metabolites [41].

The community structure change of the gut microbiota of *D. valens* feeding in 9 mg/g α-pinene concentration in phloem media after 6 h (Figure 3) may be linked to the toxic effects of the monoterpene to the microorganisms. Previous studies suggested that the amount of host defensive monoterpenes in beetles’ guts may accumulate to a very high level when feeding on substrates containing high concentration of the chemicals [38,42], and the tolerance of microorganisms to different concentrations of α-pinene is species dependent [9,31]. As the concentrations of α-pinene in *D. valens* guts in vivo were not quantified and the influence of the chemical to each gut bacteria was not assayed, this speculation need more evidence to support our claim. Meanwhile, the community structure change of gut microbiota might be due to the antifeedant effects of the defense chemical on the bark beetles (Figure 1), which would subsequently influence both nutrition and monoterpene intake of the beetle. The toxic effects of host defense chemicals like monoterpenes on bark beetles have been documented in previous studies [9,36] and other systems [43,44]. In addition to toxic substances, the available nutrition contained in food was also reported to influence the gut bacterial community structure [17,45]. We found that the relative percentage of *Erwinia* in 9 mg/g α-pinene 6 h feeding group increased compared to other groups (Table 2). The increase of *Erwinia* may attribute to the decreasing numbers of bacteria in genera *Sphingomonas* and *Rhodococcus* since the bacteria in genus *Erwinia* are sensitive to high concentration of α-pinene. The antifeedant effect of 9 mg/mL α-pinene at 6 h would preclude the chemical from entering the beetle’s intestinal track, which may promote its growth. Therefore, whether the differences of the community composition are caused by toxic activities of α-pinene to gut bacteria, antifeedant effects, decreased nutrition intake of *D. valens*, or a combination of them requires further research to verify the mechanism.

Furthermore, our evidence suggests that the influence of α-pinene on the *D. valens* gut bacterial community is not permanent and irreversible, and the structure change of *D. valens* gut microbiota of beetles feeding on 9 mg/g α-pinene in phloem media after 6 h has been recovered to a stable status after 48 h (no significance of gut community structures among 0 mg/g α-pinene 6 h, 0 mg/g α-pinene 48 h, and 9 mg/g α-pinene 48 h feeding groups) (Figure 3). A stable gut community structure is very important for insect hosts’ growth and survival, which has been proven in mammal animals and insects [46,47,48]. For example, the gut microbiota of *Pyrrhocoris apterus* is remarkably stable, which plays an important role for host nutrition [48,49]. The mating preference of *Drosophila melanogaster* could be irreversibly abolished by antibiotic treatment, which would influence its intestinal microbiota [46,50]. The functional importance of the stability of the gut bacterial community to *D. valens* need more research to explore and verify.

Our results support the hypothesis that the gut microbiota of *D. valens* is capable of helping the beetle detoxify host pine defensive chemicals. The three most abundant genera found in *D. valens* gut microbiota in our study are known to play a role in toxin metabolism (Table 2). The most predominant genus, *Sphingomonas* (Table 2), is reported to catabolize monoterpenes and other aromatic compounds [51,52,53]. The bacteria in genera *Rhodococcus* and *Burkholderia*, which also have high abundance in *D. valens* gut bacteria communities (Table 2), are reported to degrade monoterpenes, e.g., α-pinene, limonene [54,55,56]. It is also possible that other less abundant gut microbes (e.g., bacteria in genus *Pseudomonas*) help the beetle catabolize and further detoxify plant defense chemicals or perhaps other functions related to detoxification processes, such as free-radical scavenging [33,41]. In the future, we plan to conduct community metagenomic sequencing to learn about gene-centred details relevant to the detoxification of pine terpene defenses by the gut microbiota of *D. valens*.

## 4. Materials and Methods

### 4.1. Insects

Adult beetles were captured during the dispersal phase using Lindgren funnel traps baited with kairomone lure ((+)-α-pinene: (−)-β-pinene: (+)-3-carene = 1:1:1) (Aldrich, Shanghai, China) from May to June 2014. Field trapping was conducted in the Tunlanchuan Forestry Station (N 37°48′, E 111°44′, average elevation 1400 m), west of Gujiao, China. Sexes of bark beetles were distinguished by listening for stridulation produced by males [57,58]. Log bolts (≥30 cm) were cut into 0.5 m lengths and two pairs of adult beetles were introduced to each bolt after the cut ends of bolts were waxed, then bolts were placed in plastic boxes (40 cm diameter, 50 cm height) at room temperature [9,36]. Beetles from the next generation were collected as they emerged from infested bolts.

### 4.2. The Boring Lengths of D. valens at 0 and 9 mg/g Concentration of Host Defensive Compound α-Pinene

To make phloem medium, *Pinus tabuliformis* phloem was freeze-dried, ground, and autoclaved to sterilize and remove volatile monoterpenes, as described previously [7]. Six grams of agar (NewProbe, Beijing, China) was mixed with 180 mL boiling distilled water and twelve grams of ground phloem, then the corresponding amount of α-pinene was dissolved in pentane (HPLC purity; J&K Scientific) [36], and it was added into medium after cooling to about 50 °C (final concentration of α-pinene: 0, 9 mg/g, respectively) [7]. Nine milligram per gram was set as a concentration of α-pinene of host pines after beetles’ attack because the mean quantities of α-pinene range from 0.1 mg/g to 1.6 mg/g in phloem tissue of healthy tree [36], and the concentration of the chemical around beetle’s galleries would increase 5–10-folds after beetles’ attack [59]. About 3.5 mL of phloem medium was then poured into each glass tube (1 cm diameter, 5 cm height), and the glass tubes were stoppered at both ends by plastic caps and sealed with parafilm, and then phloem medium was dried for 12 h.

Adult beetles (*n* = 200) were randomly chosen and separated into two groups and weighed, and then introduced into the media individually after they had been starved for 12 h. It has been reported that the long chain of behavioral steps of bark beetles from landing to continued oviposition was divided into several phases, and the beetles walk on the logs under attack within the first day, and then they would complete nuptial chamber construction in the next 1–2 days [60]. Therefore, 6 and 48 h was selected as two time-points. After 6 h feeding at room temperature, the boring lengths of half of the beetles in each group were measured (40 beetles in 0 mg/g α-pinene concentration in phloem media, 47 beetles in 9 mg/g α-pinene concentration in phloem media) (AL 204, Mettler Toledo, Inc., Shanghai, China), and 13 beetles (6 beetles in 0 mg/g α-pinene concentration in phloem media, 7 beetles in 9 mg/g α-pinene concentration in phloem media) were randomly chosen for gut microbiota analysis. After 48 h feeding, the boring lengths of the remaining beetles were measured individually (39 beetles in 0 mg/g α-pinene concentration in phloem media, 39 beetles in 9 mg/g α-pinene concentration in phloem media), and 12 beetles (5 beetles in 0 mg/g α-pinene concentration in phloem media, 7 beetles in 9 mg/g α-pinene concentration in phloem media) were randomly chosen for gut microbiota analysis. Boring lengths for those beetles that failed to enter the media were scored as zero and dead beetles were discarded.

### 4.3. DNA Extraction, PCR, Pyrosequencing, and Sequence Processing

The beetles from 0 mg/g α-pinene phloem media and 9 mg/g α-pinene phloem media after 6 h (0 mg/g α-pinene 6 h feeding group and 9 mg/g α-pinene 6 h feeding group) and 48 h (0 mg/g α-pinene 48 h feeding group and 9 mg/g α-pinene 48 h feeding group) were dissected, and then the bacteria genomic DNA of each insect gut sample was extracted by using a TIANamp Bacteria DNA kit (TianGen, Beijing, China) according to the manufacturer’s instructions, respectively. The PCR reactions were carried out in a 20 μL of solution containing 10 ng of DNA, 1 μL of 10 μM of each primer, 2 μL of 2.5 mM dNTPs, 0.3 μL Fastpfu polymerase (Transgene, Beijing, China), and 4 μL 5× Fastpfu buffer. The amplifications were performed in an ABI GeneAmp^®^ 9700 thermal cycler (Applied Biosystems, Foster City, CA, USA) with an initial denaturation step at 95 °C for 10 min followed by 30 cycles of annealing and extending (each cycle occurred at 95 °C for 30 s followed by 55 °C for 30 s and an extension step at 72 °C for 45 s) and the final extension at 72 °C for 10 min using 16S rRNA primers 341F (5′-CCTAYGGGRBGCASCAG-3′) and 806R (5′-GGACTACHVGGGTWTCTAAT-3′) [61]. The final PCR products were analyzed by electrophoresis in 1.5% agarose gel followed by staining with ethidium bromide and visualization under ultraviolet light. The purified amplicons were pyrosequenced on an Illumina platform (Illumina MiSeq PE250, Illumina, CA, USA).

Paired-end reads were assembled with FLASH (V1.2.7, Available online: www.ccb.jhu.edu) and low-quality reads were filtered using the QIIME (Quantitative Insights Into Microbial Ecology) software packages (V1.9.0, Available online: www.qiime.org) with default parameters [62]. Chimeras were checked and removed with UCHIME [63] and qualified sequences were clustered into Operational Taxonomic Units (OTUs) at 97% sequence similarity with a UPARSE algorithm [64]. The representative OTU was selected based on the most abundant sequence in each OTU, and then taxonomic identification was performed using the RDP classifier [65] algorithm implemented in QIIME and using the Greengene database under a confidence threshold of 80% (Available online: http://greengenes.secondgenome.com) [66].

### 4.4. Statistical Analysis

In comparisons of the boring length of beetles between 0 and 9 mg/g α-pinene feeding groups, means of cases were tested using independent *t*-test or Mann–Whitney *U*-test, depending on the results of the test of normality and homogeneity of variance. Data were analyzed using SPSS 12.0 (SPSS Inc., Chicago, IL, USA) for Windows, and figures were drawn using Origin 8.5 (Origin Lab Corporation, Northampton, MA, USA).

For MiSeq data analysis, rarefaction curves were estimated using the “alpha_rarefaction.py” script in QIIME to test whether the sequencing efforts adequately represented the bacterial diversity within each sample. Two richness estimators (the abundance-based coverage estimator (ACE) and a nonparametric richness estimator based on distribution of singletons and doubletons (Chao1)) and two diversity indices (Shannon and Simpson index) were calculated for the samples using the “alpha_diversity.py” script in QIIME. The diversity indices of four groups and the relative abundances of different genera were compared using One-way ANOVA test followed by Bonferroni test (equal variances) or One-way Brown-Forsythe’s ANOVA test followed by Dunnett’s T3 test (unequal variances). Non-metric multidimensional scaling (NMDS) was used to visualize the phylogenetic distance (Jaccard similarity) between the bacterial communities from different samples. Composition differences were tested using ANOSIM with 10,000 permutations using PAST software [67,68]. The representative sequences of all OTUs were used to construct neighbor-joining trees. The phylogenetic tree together with sample sequence abundance data were used for weighted Unifrac PCoA (principal coordinate analysis) which considers both relative abundance and different branch lengths in a tree, through the online Fast Unifrac program [69]. A Permutational Multivariate Analysis of Variance based on the weighted UniFrac distance (PERMANOVA, “PermanovaG” function in the “GUniFrac” package of R) was used to test for differences in community composition between four sample groups [70].

## 5. Conclusions

In summary, our results suggested that 9 mg/g α-pinene concentration significantly inhibited feeding behavior of *D. valens* and altered its gut microbial community structure after 6 h. The inhibition of feeding behavior from 9 mg/g α-pinene in diets ceased after 48 h. No significant differences of the bacterial communities were observed between the beetles in 0 and 9 mg/g α-pinene concentration in phloem media after 48 h. We found that both the inhibition of the feeding behavior of *D. valens* and the disturbance to its gut bacterial communities by high concentration of host defensive α-pinene in phloem media after 6 h were eliminated soon after 48 h, which suggests the quick adaptation of both the beetle and its gut microflora to high concentration of host defensive chemical.

## Figures and Tables

**Table 1:** Comparison of diversity indices (Mean ± SEM) among different groups. Different superscript letters indicate significant differences across treatments (*p* < 0.05); OTUs, Operational Taxonomic Units.

Index	0 mg/g α-Pinene 6 h	9 mg/g α-Pinene 6 h	0 mg/g α-Pinene 48 h	9 mg/g α-Pinene 48 h
Number of OTUs	273.8 ± 28.62	306.6 ± 30.14	277.0 ± 26.36	268.1 ± 17.76
ACE diversity	455.4 ± 57.56	527.5 ± 54.89	441.2 ± 62.43	402.4 ± 33.63
Chao diversity	443.0 ± 49.51	532.7 ± 52.08	449.8 ± 64.44	403.1 ± 35.85
Shannon diversity (H)	1.62 ± 0.09 ^b^	1.86 ± 0.02 ^b^	1.61 ± 0.14 ^b^	1.52 ± 0.05 ^a^
Simpson diversity	0.49 ± 0.02 ^a,b^	0.60 ± 0.00 ^b^	0.48 ± 0.02 ^a,b^	0.46 ± 0.02 ^a^

**Table 2:** Shared genera among groups. Identity of the genera shared by four different groups and their average abundance (mean ± SEM) within each group are displayed. The genera that had an abundance of at least 0.1% of the total number of reads in at least one sample were present. The sum of all taxa present in the table within each group is shown in the last row of the table. Different superscript letters indicate significant differences across treatments (*p* < 0.05).

Phylum	Phylogenetic Group (Genus)	0 mg/g α-Pinene 6 h %	9 mg/g α-Pinene 6 h %	0 mg/g α-Pinene 48 h %	9 mg/g α-Pinene 48 h %
Actinobacteria	*Rhodococcus*	20.42 ± 0.86	17.15 ± 1.99	20.48 ± 2.40	21.58 ± 0.99
	*Salinibacterium*	0.93 ± 0.20	1.00 ± 0.17	1.17 ± 0.29	1.19 ± 0.20
Proteobacteria	*Burkholderia*	1.05 ± 0.16 ^a,b^	0.69 ± 0.12 ^a^	1.22 ± 0.09 ^a,b^	1.33 ± 0.19 ^b^
	*Erwinia*	5.80 ± 1.96 ^b^	24.41 ± 5.74 ^a^	4.05 ± 2.30 ^b^	2.37 ± 1.17 ^b^
	*Pseudomonas*	0.26 ± 0.06	0.10 ± 0.03	0.29 ± 0.09	0.36 ± 0.12
	*Sphingomonas*	70.35 ± 1.62 ^b^	55.85 ± 4.26 ^a^	71.69 ± 1.30 ^b^	72.24 ± 1.56 ^b^
	Sum	98.81 ± 0.00	99.20 ± 0.00	98.91 ± 0.00	99.06 ± 0.00

## Supplementary Materials

Supplementary materials can be found at www.mdpi.com/1422-0067/17/10/1734/s1.
