# Supplementary material for: Pine Defensive Monoterpene α-Pinene Influences the Feeding Behavior of Dendroctonus valens and Its Gut Bacterial Community Structure
Source: Int J Mol Sci. 2016 Nov 1;17(11):1734. doi: 10.3390/ijms17111734 (PMC5133772; doi:10.3390/ijms17111734)
Supplement: Supplementary file 1 [file ijms-17-01734-s001.zip › ijms-140937-Supplementary Table S1.pdf]

Supplementary Materials: Pine Defensive Monoterpene α-Pinene Influences the Feeding Behavior of *Dendroctonus valens* and Its Gut Bacterial Community Structure

Letian Xu, Zhanghong Shi, Bo Wang, Min Lu and Jianghua Sun

Table S1. Results of ANOSIM comparing of all bacterial communities. * $p < 0.05$.

Treatments	0 mg/mL α-Pinene 6 h	0 mg/mL α-Pinene 48 h	9 mg/mL α-Pinene 6 h
0 mg/mL α-pinene 6 h			
0 mg/mL α-pinene 48 h	0.491		
9 mg/mL α-pinene 6 h	0.021 *	0.004 *	
9 mg/mL α-pinene 48 h	0.242	0.452	0.001 *
